# High Adult Sex Ratios and Risky Sexual Behaviors: A Systematic Review

**DOI:** 10.1371/journal.pone.0071580

**Published:** 2013-08-13

**Authors:** Cedric H. Bien, Yong Cai, Michael E. Emch, William Parish, Joseph D. Tucker

**Affiliations:** 1 University of North Carolina Project-China, Guangzhou, China; 2 University of North Carolina at Chapel Hill, Chapel Hill, North Carolina, United States of America; 3 University of Chicago, Chicago, Illinois, United States of America; University of Missouri Kansas CIty School of Medicine, United States of America

## Abstract

**Background:**

Thirty-four countries worldwide have abnormally high sex ratios (>102 men per 100 women), resulting in over 100 million missing women. Widespread sex selective abortion, neglect of young girls leading to premature mortality, and gendered migration have contributed to these persistent and increasing distortions. Abnormally high adult sex ratios in communities may drive sexually transmitted disease (STD) spread where women are missing and men cannot find stable partners. We systematically reviewed evidence on the association between high community sex ratios and individual sexual behaviors.

**Methods and Findings:**

Seven databases (PubMed, Web of Science, Embase, Scopus, The Cochrane Database of Systematic Reviews, Sociological Abstracts, and PopLINE) were searched without restrictions on time or location. We followed PRISMA guidelines and evaluated quality according to STROBE criteria. 1093 citations were identified and six studies describing 57,054 individuals were included for review. All six studies showed an association between high community sex ratios and individual sexual risk behaviors. In high sex ratio communities, women were more likely to have multiple sex partners and men were more likely to delay first sexual intercourse and purchase sex. Only two studies included STD outcomes.

**Conclusions:**

High community sex ratios were associated with increased individual sexual risk behavior among both men and women. However, none of the studies examined unprotected sex or appropriately adjusted for gendered migration. Further studies are needed to understand the effect of community sex ratios on sexual health and to inform comprehensive STD control interventions.

## Introduction

117 million women are now missing worldwide, according to United Nations Population Fund estimates [1]. These missing women and corresponding “surplus men,” men who cannot find stable partners, reflect increasingly distorted adult sex ratios (number of men per 100 women). Although normal population sex ratios tend to fall between 94 to 102 men per women [2], China (106) and India (108) have abnormally high sex ratios [3]. While China and India account for roughly 80 million of missing women [1], [4], 32 other nations clustered in Asia and the Middle East also have abnormally high sex ratios (>102 men per 100 women) [3], [5].

Abnormally high adult sex ratios result from sex selective abortions, neglect of young girls leading to premature mortality, and gendered migration. Sex selective abortions have become so widespread that the global sex ratio at birth has increased from 105 to 107 [6]–[8]. Excess mortality of girls due to infanticide and neglect remains a persistent problem in many regions [5], [7], [9]. Distorted local sex ratios are further skewed because of male predominant migration to urban areas in search of brides and jobs [10]–[12].

High sex ratios establish communities where surplus men cannot form stable partnerships with women, potentially driving risky sexual behaviors that accelerate transmission of sexually transmitted diseases (STDs). Increased STD burden in high sex ratio communities may be due to increased unsafe commercial sex [10], [13], [14], forced sex [15], increased homosexual sex [16], [17], and wife trafficking [18]–[20]. High adult sex ratios are also associated with premature male mortality [21], and increased depression and suicidality [22], [23]. However, it has also been speculated that high sex ratios decrease male sexual risk behavior, because men have fewer potential female sexual partners [24], [25]. High adult sex ratios may put women at increased STD risk, because women may have more sexual partners due to greater partner availability.

The potential for high adult sex ratios to drive STD transmission has been previously hypothesized [19], [26]–[29], but there is a lack of empirical data on the effect of high sex ratios on sexual risk behavior and STD transmission. Given the increasing number of surplus men worldwide entering marriage markets, sex ratios are a crucial variable to consider in explaining sexual risk behavior. The purpose of this study is to systematically review the association of high adult sex ratios on individual sexual risk behaviors and STD biomarkers.

## Methods

We conducted a literature search through 30 August 2012 of articles that addressed the association between adult community sex ratios and individual sexual risk behaviors and STD biomarkers. We searched abstracts using combinations of the key words *surplus men, forced bachelor, sex ratio, male-female ratio,* or *gender ratio,* and *HIV/AIDS, std, sexual behavior, sexually transmitted disease, risk behavior, sex workers,* or *commercial sex.* Our PubMed search terms are available in Text S1. We searched PubMed, Web of Science, Embase, Scopus, The Cochrane Database of Systematic Reviews, Sociological Abstracts, and PopLINE electronic databases for articles and abstracts restricted to human populations.

Abstracts were checked for potential relevance, and had to meet the following criteria: only quantitative, English-language population-based research studies; sex ratios measured across multiple partner markets (a community unit defined by each study); and reported individual sexual risk outcomes (behaviors or biomarkers). In order to conduct a comprehensive global systematic review, no limits were placed on study date, location, race or ethnicity of study participants, or the definition of sex ratio. Partner markets were defined as discrete geographic units in which individuals were more likely to find a stable partner. Behavioral outcomes included in the study were multiple sex partners, sex with commercial sex workers, forced sex, premarital sex, recent sex, and self-reported sexually transmitted disease. Biomarker outcomes included a positive test for any sexually transmitted disease. We excluded studies conducted in settings with primarily low sex ratios (e.g., due to male out-migration or imprisonment) and studies that were not population-based. Outcomes related to health seeking behaviors, mental health, or unrelated to sexual behavior were excluded. Case studies, qualitative studies, and ecological studies were excluded.

We used PRISMA guidelines to identify and exclude studies (Text S2). Two independent reviewers (CB and JT) analyzed full-text articles for inclusion in the review. The reference sections of the remaining articles were then searched to identify other studies that met our inclusion criteria. Source data were reviewed (by JT and CY) and data were abstracted into tables. We contacted study authors to retrieve missing study data. Effect size was measured according to parent data, as either the coefficient or odds ratio. In some cases, statistical significance was converted to a p-value. We used adjusted odds ratios in studies with both crude and adjusted data.

We graded the quality of each study using the STROBE reporting criteria for cross-sectional studies (Table S1) [30]. We did not conduct a meta-analysis due to lack of standardized reporting of sex ratio measures and sexual risk outcomes.

## Results

**Figure 1:**
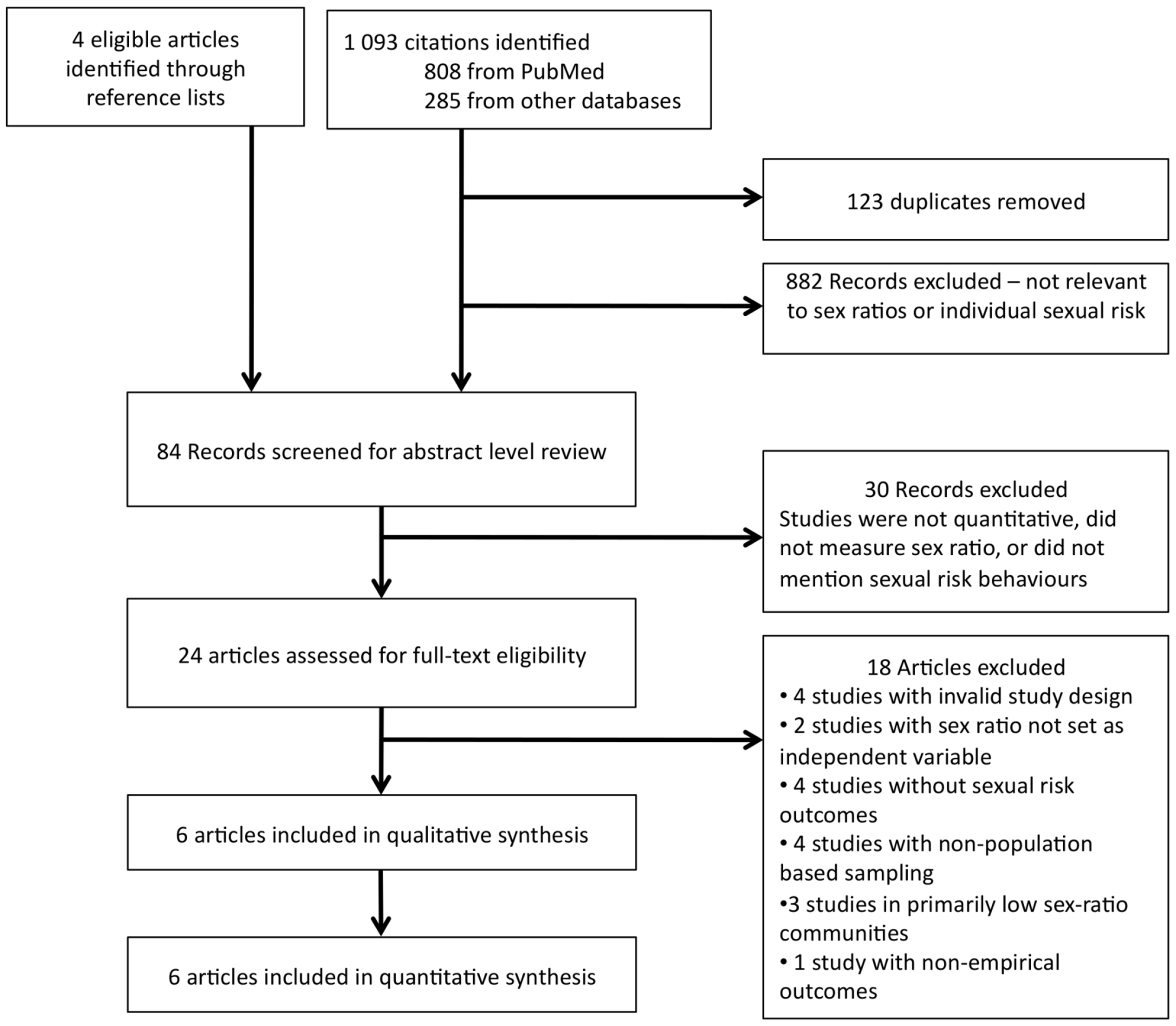
Study Selection algorithm according to PRISMA guidelines.

Our search yielded a total of 1093 studies (Figure 1). Most initial citations were excluded because they did not measure adult community sex ratios or examined sexual risk outcomes. 24 abstracts were retrieved for full-text review and six studies were included in the review. Reasons for exclusion of the remaining 18 studies were: invalid study design (n = 4), sex ratio not set as independent variable (n = 2), no sexual risk outcomes assessed (n = 4), non-population based sampling (n = 4), studies occurred in primarily low sex-ratio communities (n = 3), and modeling studies (n = 1) All studies were retrospective cross-sectional surveys.

Six studies were included in this review describing 57,054 individuals. Three studies were conducted in high-income countries [31]–[33] and three studies in middle-income countries [15], [24], [25] (Table 1). All six studies used data from national, population-representative, behavioral surveys of adults or adolescents. Study sample size ranged from 468 to 33,695 individuals. None of the studies distinguished between protected and unprotected sex. Two studies included STD biomarker data, which in both cases examined gonorrhea, chlamydia, and trichomonas [15], [24]. Five of the six studies had sex-specific data with two studies reporting male data [24], [25] and three studies reporting female data [15], [31], [32]. Five studies used multi-level analysis methods to assess community and individual level data [15], [24], [25], [31], [32], and one study did not adjust for individual level observations [33]. Five studies adjusted for age [15], [24], [25], [31], [32] and three studies adjusted for education. Adjustment for other individual level factors such as race, income, and marital status varied between studies. Using STROBE criteria for cross-sectional studies, we found substantial variation in reporting quality among studies.

**Table 1:** Overview of studies of community sex ratios and STD risk.

Study	Date of Study	Location	Sample size	Study population	Number of partnermarkets	Partner market	Quality Assessment^a^
Billy et al 1994 [31]	1982	United States	1,852	Adolescent women ages15–19	Not reported	U.S. census tract	59% (19/32)
Browning et al 2003 [32]	1990, 1994–1997	Chicago, United States	468	192 men, 276 womenages 18–59	342	Neighborhood clusters	63% (20/32)
Smith et al 2006 [33]	2001–2002	Australia	18,647	Australians aged 16–59	200	Australian statistical subdivisions	59% (19/32)
South et al 2010 [24]	2000	China	1,023	Non-migrant Chinese menages 20–44	37	County or county-equivalent	84% (27/32)
South et al 2012 [25]	2005–06	India	33,695	Non-migrant men ages15–39	2,497	Community^b^	72% (23/32)
Trent et al 2012 [15]	2000	China	1,369	Chinese women ages20–44	37	County or county-equivalent	63% (20/32)

a Quality assessment represents the percentage and summary score of the number of STROBE criteria reported, over the number of total number of criteria.

b Community definition was not given in this study.

Definitions of adult sex ratios, migration, and partner markets substantially differed between studies. Although the adult sex ratio is typically reported as the number of men per 100 women of a certain age, two studies reported the number of women per 100 men. These measures were not converted into standard measures of sex ratios in order to preserve apparent effect sizes from the studies. None of the studies included individual-level data for migration, although two of the studies included migrants in community-level census data but excluded migrants in the study sample [15], [24]. Partner markets were generally defined at the census tract or county level, but in one study the community size was not specified [25].

All six studies found an association between high sex ratios and increased sexual risk outcomes (Table 2). Among the three studies that had individual male data [24], [25], [32], one study found a positive association between high sex ratios and number of short-term sexual partners in the past year (b = 0.38, p<0.01) [32]. Two studies found that men in high sex ratio communities in India (b = −0.008, p<0.05) [25] and China (b = −.20, p>0.10) [24] were more likely to purchase sex. Three studies had individual female data [15], [31], [32]. One study conducted in the United States found that in high sex ratio communities, adolescent women had an increased likelihood of having had sex (b = 0.145, p>0.20 nonblack women; b = 3.398, p<0.05 black women) and more frequent sex (b = 0.926, p<0.05 nonblack women, b = 3.956, p<0.05 black women) [31]. The study also found that black adolescent women in high sex ratio communities spent a higher proportion of the year being sexually active (b = 0.637, p<0.05). One study in a single city in the United States found that women in high sex ratio communities reported fewer short-term sex partners (b = 0.039, p>0.10), despite their male cohorts reporting more sex partners [32]. One study in China found that in high sex ratio communities, Chinese women had an increased likelihood of having had sex in the past year (b = 0.038, p<0.01) and test positive for an gonorrhea, chlamydia, or trichomonas infections (b = 0.022, p<0.05) [15]. Chinese women were also more likely to have had forced sex (b = 0.018, p>0.10) and premarital sex (b = 0.017, p>0.10) in high sex ratio communities, although these associations were not statistically significant.

**Table 2:** Summary of results from included studies, focused on examining the relationship between high sex ratio communities and STD risk.

Study	Sex ratiorange^a^	Biomarker outcome	Behavioral outcome	Effect size (Coefficientor Odds Ratio)	Statisticalsignificance	Association betweenhigh sex ratiosand male sexualrisk?	Association between high sex ratios and female sexual risk?
Billy et al1994 [31]	Not stated		Ever had sexualintercourse	0.145 (nonblacks),3.398 (blacks)	p>0.20 (nonblacks),p<0.05 (blacks)	Not assessed	Positive
			Coital frequency	0.926 (nonblacks),3.956 (blacks)	p<0.05 (nonblacks),p<0.05 (blacks)	Not assessed	Positive
			Proportion of monthsspent sexually active	0.637 (blacks)	p<0.05 (blacks)	Not assessed	Positive
Browninget al2003 [32]	M = 88.9, SD = 15.2		Short-term sex partnersin the last year	0.38 (men), −0.039(women)	p<0.01 (men),p>0.10 (women)	Positive	Not significant^b^
Smith et al 2006 [33]	Not stated		No partners	OR = 0.917, 95% CI(0.894,0.941)		Positive	Positive
			Two partners	OR = 0.981, 95% CI(0.955,1.007)		Not significant	Not significant
			Three or more partners	OR = 0.954, 95% CI(0.925,0.984)		Negative	Negative
Trent et al2012 [15]	Min = 80, max = 120		Sexual intercourse inpast year	0.038	p<0.01	Not assessed	Positive
			Forced sex	0.018	p>0.10	Not assessed	Not significant
			Premarital sex	0.017	p>0.10	Not assessed	Not significant
		Gonorrhea, chlamydia, trichomonas STI		0.022	p<0.05	Not assessed	Positive
**Studies with sex ratio defined as women per 100 men**							
South et al2010 [24]	M = 94.2, SD = 6.22		Premarital sex	0.015	p<0.05	Negative	Not assessed
			Commercial sex	−0.20	p>0.10	Not significant	Not assessed
		Gonorrhea, chlamdyia, or trichomonas STI		0.031	p<0.05	Negative	Not assessed
South et al2012 [25]	M = 133.2, SD = 23.99		Premarital sex	0.002	p>0.10	Not significant	Not assessed
			Two or more partners	0.002	p>0.10	Not significant	Not assessed
			Commercial sex	−0.008	p<0.05	Positive	Not assessed
			Self-reported STI	0.001	p>0.10	Not significant	Not assessed

a Sex ratio ranges reported as Mean (M) and Standard Deviation (SD).

b Association is summarized as “not significant” if p>0.10.

Two studies found a concurrent decrease in some sexual risk outcomes and increase in other sexual risk outcomes [24], [33]. One Australian study found that in high sex ratio communities, adults were less likely to be sexually inactive (OR = 0.917, 95% CI 0.894,0.941), but were also less likely to have had at least three sexual partners compared with one partner (OR = 0.954, 95% CI 0.925,0.984) [33]. This study did not differentiate between male and female sexual behaviors. A study of Chinese men found that although men in high sex ratio communities were more likely to have had commercial sex in high sex ratio communities, they were less likely to have had premarital sex (q = 0.015, p<0.05), or receive a positive test for gonorrhea, chlamydia or trichomonas infections (q = 0.031, p<0.05) [24].

Few of these studies examined mechanisms linking the connection between high community sex ratio and individual behavior. Two studies examined purchasing of sex among men [24], [25], and one study examined forced sex among women [15]. None of the studies examined increased partner concurrency, unprotected sex, intimate partner violence, or wife trafficking.

## Discussion

**Figure 2:**
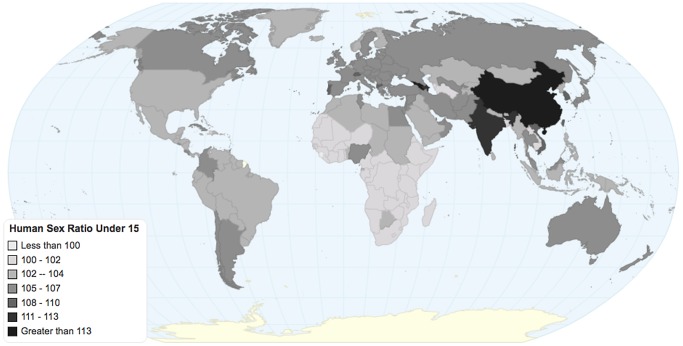
Human Sex Ratio Under 16. Sex ratio is defined as the number of men per 100 women in populations less than 16 years old. Source: Sex Ratio: Central Intelligence Agency 2011, The World Factbook 2011, Washington DC.

High adult sex ratios are increasingly common in many parts of the world, but the impact of these global demographic changes on individual sexual behaviors is poorly understood. The proportion of men older than 25 years old who fail to marry in China will nearly triple in the next twenty years, even if sex ratio trends reverse now [14]. This emerging cohort is increasingly sexually active (Figure 2). Most studies investigating the association of high community sex ratios on individual risk behavior are limited to small community samples [34]–[38], or do not include both individual- and community-level data [16], [39], [40]. We found evidence from two population-based studies that high sex ratios were associated with increased sexual risk behaviors among women. Our data suggest that women in high sex ratio communities were more likely to have ever had sex, have more frequent sex, and have an STD. The relationship between high sex ratios and male sexual behaviors is less clear. Although men may have fewer sexual partners in high sex ratio communities, more men may purchase sex when faced with a relative dearth of women. Few studies examined mechanisms linking community sex ratios and individual sexual behaviors.

Our review found limited evidence that high sex ratios were associated with increased sexual risk behavior among women. One study in China [15] and one in the United States [31] found that women in high sex ratio communities were more likely to have had recent sex. One study found women in high sex ratio communities were more likely to have an STD and were more likely to have had forced sex [15]. These data are inconsistent with hypotheses that female empowerment would increase and gender-based violence would decrease in communities with fewer men [41], [42]. Although greater numbers of male partners may lead to better marriage prospects for women, women in high sex ratio communities also face greater structural violence and gender inequities that may increase sexual risk behaviors [18]. One study conducted in a single city of the United States did not find a significant association between high community sex ratios and number of sexual partners among women, even though men reported greater number of sexual partners [32]. One possible explanation for this finding is that sexual networks may extend beyond the scope of the single-city survey, or that highly sexually active women were not captured by the study sampling methods.

Our review suggests increased sexual risk behaviors among men in high sex ratio communities as well. Two studies found that men in high sex ratio communities delay first sexual intercourse, but have an increased risk of purchasing sex, compared to men in normal sex ratio communities [24], [25]. These findings support the hypothesis that as men in high sex ratio communities have fewer opportunities to find stable female sex partners, they increasingly turn to less stable commercial sex partners. Another study also found that adults in high sex ratio communities were more likely to be sexually active but had fewer partners. [33] One study found an association between higher sex ratios and increased number of sex partners among men [32].

**Figure 3:**
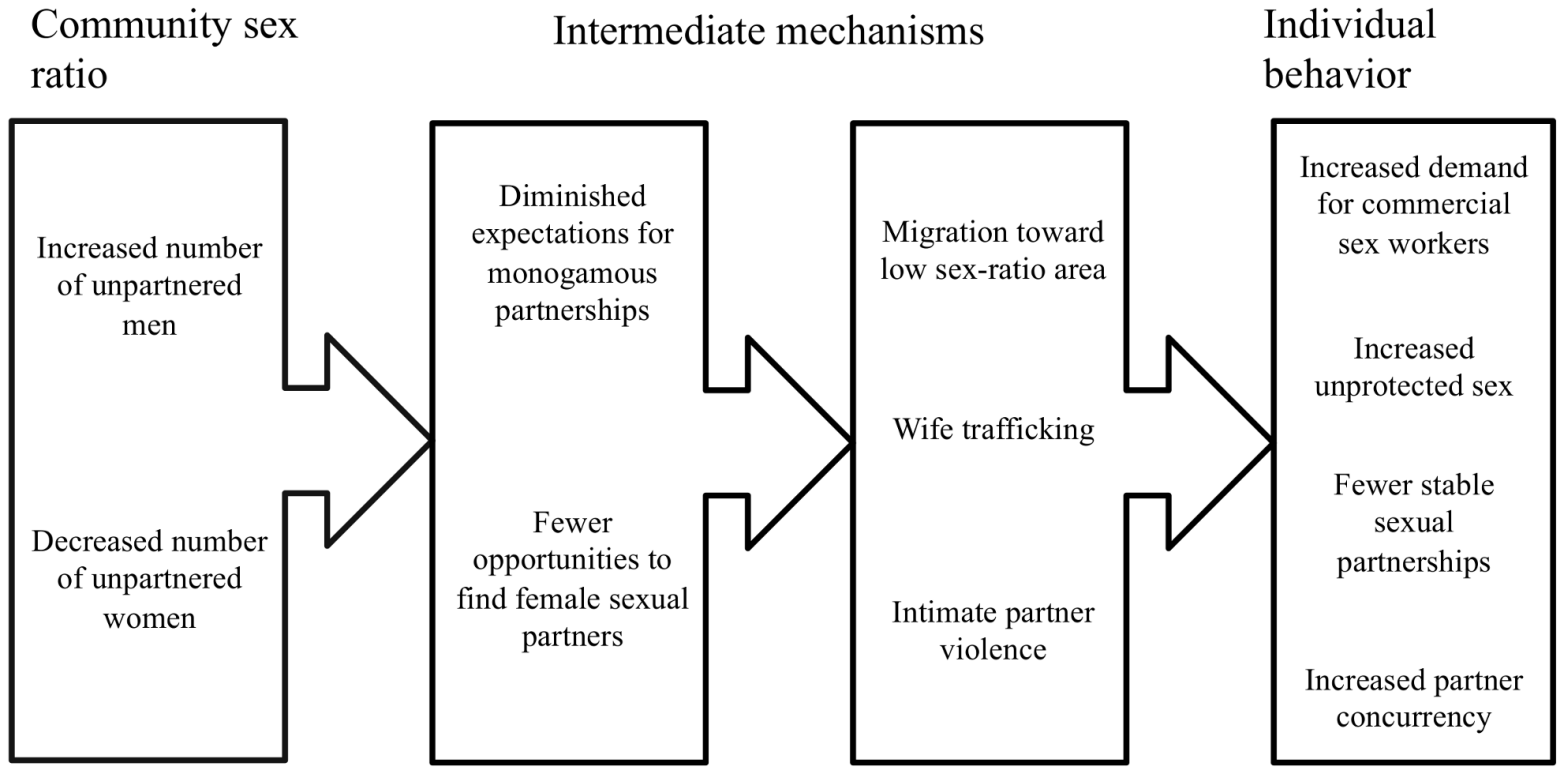
Potential mechanisms linking high community sex ratios and increased sexual risk behaviors.

Understanding the mechanisms that link community sex ratios and individual risk can guide risk-reduction strategies, from community-level to individual-level interventions. However, we found little evidence that linked the impact of community sex ratios and individual sexual risk behaviors (Figure 3). The only mechanisms that studies investigated were commercial sex use among men [24], [25] and forced sex among women [15]. Increased purchasing of sex has been documented in other contexts that examine communities with a surplus of men [34], [43]. The association between high sex ratio communities and forced sex in China requires further investigation [15], particularly in light of increased violence [44], [45] and reports of wife trafficking [20] in high sex ratio communities. One theoretical framework, called the demographic-opportunity theory, holds that the likelihood of cross-sex interactions increases when one sex is faced with a relative surplus of the opposite sex [37]. Distorted community sex ratios may increase social permissiveness and lead to increased sexual partner concurrency [31], [32]. Increased surplus men in rural areas may encourage male migration toward urban areas where men may have greater access to commercial sex [29].

Migration of surplus men away from high sex ratios is a critical issue because it may inadvertently increase sex ratios in destination communities. However, none of the studies examined gendered migration as either a potential driver of increased sex ratios or a potential cause of individual risk behavior. One study of migrant communities in North Carolina with high sex ratios found an increase in commercial sex use among in communities with fewer women [34]. The potential for rural to urban migration to increase sexual risk has been documented in several settings, where decreased social stability may also contribute to increased sexual risk among men [12], [46], [47].

Despite our comprehensive search strategy, our review highlights the relatively limited literature on community sex ratios and sexual health. The reporting quality of some of the studies was poor. One study presented only data that did not differentiate male and female sexual behaviors [33]. One study did not define community size [25] and another did not include the number of communities included in the study [31]. One study was conducted in a single city in the United States [32]. Further research is needed to examine if sexual networks extend beyond communities, particularly in areas with high sex ratios. None of the studies distinguished between unprotected and protected sex, a critical behavioral issue. Most studies included only limited data on sexual experience and only two studies examined STD biomarker endpoints [15], [24]. In capturing sexual behavior and STD outcomes, none of the studies captured data on whether sexual partners originated from the same community. Given the increasing availability of sensitive and specific point-of-care tests for HIV and syphilis [48], future studies should aim to include both behavioral and biomarker endpoints. None of the studies included in the review examined individual migration status, introducing selection bias of study participants. The transience of migrant men creates challenges for population-based sampling, and two studies in our review excluded migrant men or men who had recently changed residence [24], [25].

Our review has several limitations. First, meta-analysis was not possible due to substantial variation in study methodology. Although sex ratios are commonly reported as number of males per 100 females, two studies reported sex ratios as the number of females per 100 males. Variations in study definition of sex ratio and outcomes measures did not permit comparisons of effect size. In order to broaden the scope of the review, we did not place any restrictions on time or location of included studies. Nevertheless, our study included data from only four countries, introducing the possibility of publication and location bias. Three of the included studies were conducted in countries with high sex ratios, but other nations, particularly those in Asia, also have abnormally high sex ratios. Future studies in these settings are warranted. Increased understanding of the mechanisms that drive sexual risk behavior in high sex ratio communities will strengthen comparability between study settings. We did not include studies where sex ratios are predominately low, a demographic phenomenon noted in African-American communities, communities that experience net out-migration, and nations in Eastern Europe [35], [36], [49]. Finally, we cannot infer causality based on the cross-sectional data presented.

Profound demographic changes will fundamentally shift population structures across the globe. Sex ratios will continue to rise in many regions of the world, but their effect on human health, particularly sexual health, remains poorly described. Our review found evidence that high community sex ratios may be associated with increased sexual risk behaviors among both men and women. More epidemiology research, especially among men, is needed to understand the mechanisms driving this association. In addition, prospective studies that include individual STD and migration data are warranted to further assess the findings in this review. Understanding the impact of community sex ratios on sexual health will inform structural STD control interventions, which can better target populations at increased STD risk.

## Supporting Information

Table S1 **STROBE reporting criteria for cross-sectional studies (full-text).**
(DOCX)

Text S1 **PubMed Search Strategy.**
(DOCX)

Checklist S1 **PRISMA Checklist.**
(DOC)
