# Supplementary material for: High Adult Sex Ratios and Risky Sexual Behaviors: A Systematic Review
Source: PLoS One. 2013 Aug 13;8(8):e71580. doi: 10.1371/journal.pone.0071580 (PMC3742505; doi:10.1371/journal.pone.0071580)
Supplement: Text S1 — PubMed Search Strategy. (DOCX) [file pone.0071580.s002.docx]

Our search string (limited to humans) was: "sex ratio"[MeSH Terms] OR ("sex ratio"[MeSH Terms] OR ("sex"[All Fields] AND"ratio"[All Fields]) OR "sex ratio"[All Fields]) AND "risk-taking"[MeSH Terms] OR ("risk-taking"[MeSH Terms] OR "risk-taking"[All Fields] OR ("risk"[All Fields] AND "taking"[All Fields]) OR "risk taking"[All Fields]) OR risky[All Fields] AND ("sexual behavior"[MeSH Terms] OR ("sexual"[All Fields] AND "behavior"[All Fields]) OR "sexual behavior"[All Fields])
